# Discovery of Diagnosis Pattern of Coronary Heart Disease with Qi Deficiency Syndrome by the *T*-Test-Based Adaboost Algorithm

**DOI:** 10.1155/2011/408650

**Published:** 2011-08-21

**Authors:** Huihui Zhao, Jianxin Chen, Na Hou, Peng Zhang, Yong Wang, Jing Han, Qin Hou, Qige Qi, Wei Wang

**Affiliations:** ^1^Beijing University of Chinese Medicine, Beijing 100029, China; ^2^Beijing Hospital of Traditional Chinese Medicine, Beijing 100010, China; ^3^Beijing Document Service, Beijing, 100036, China

## Abstract

Coronary heart disease (CHD) is still the leading cause of death for adults worldwide. Traditional Chinese medicine (TCM) has a history of 1000 years fighting against the disease and provides a complementary and alternative treatment to it. Syndrome is the core of TCM diagnosis and it is traditionally diagnosed based on macroscopic symptoms as well as tongue and pulse recognitions of patients. Establishment of the diagnosis method in the microcosmic level is an urgent and major problem in TCM. The aim of this study was to establish characteristic diagnosis pattern for CHD with Qi deficiency syndrome (QDS). Thirty-four biological parameters were detected in 52 patients having unstable angina (UA) with or without QDS. Then, we presented a novel data mining method, *t*-test-based Adaboost algorithm, to establish highest prediction accuracy with the least number of biological parameters for UA with QDS. We gained a pattern composed of five biological parameters that distinguishes UA with QDS patients from non-QDS patients. The diagnosis accuracy of the patterns could reach 84.5% based on a 3-fold cross validation technique. Moreover, we included 85 UA cases collected from hospitals located in the north and south of China to further verify the association between the pattern and QDS. The classification accuracy is 83.5%, which keeps consistent with the accuracy obtained by the cross-validation technique. The association between a symptom and the five biological parameters was established by the data mining method and it reached an accuracy of *∼*80%. These results showed that the *t*-test-based Adaboost algorithm might be a powerful technique for diagnosing syndrome in TCM in the context of CHD.

## 1. Introduction

Coronary heart disease (CHD) remains the single leading cause of death for adults worldwide [1]. Effective prevention and therapy for CHD poses a major challenge to the entire medical community. Traditional Chinese medicine (TCM) has fought against CHD and its related diseases for more than 1000 years and has accumulated thousands of prescriptions as well as clinical literatures. Therefore, more and more patients all over the world take TCM as a complementary and alternative avenue to treat CHD.

TCM is a medical system with at least 3000 years of uninterrupted clinical practice and it has the advantage of collecting macroscopic information (including symptoms, tongue and pulse recognition) of a patient for diagnosis, while syndrome is the core of diagnosis and target of herbal remedy in TCM. Nowadays, syndrome in TCM has been always studied in the context of a specific disease or biomedical condition and several literatures have demonstrated that syndromes are significantly associated with diseases [2, 3]. However, the biological basis of a syndrome in the context of a disease is rarely studied. The issue is significantly critical since it not only establishes a diagnosis avenue in microcosmic level, but also divides the disease into several subtypes and provides a basis for individual therapy.

Unstable angina (UA) is a type of CHD. It describes a biomedical condition that is intermediate between myocardial infarction (MI) and the more chronic state of stable angina. UA is now a heavy burden on the society and families in both industrialized and developing countries. So UA presents a better example and context for investigating the diagnosis method and biological basis of syndromes in TCM.

Qi deficiency syndrome (QDS) is an important syndrome in the UA with the frequency *∼*70%. The symptoms as well as related tongue and pulse information that serve as diagnosing QDS could be easily distinguished from UA patients, such as spiritlessness, hypodynamia, short breath, thin and fatty tongue as well as deep and thread pulse. The macroscopic phenotypes do have corresponding changes in the microcosmic levels. Therefore, establishing diagnosis method of syndromes in the microcosmic level plays a key role in understanding biological basis of them and their associated macroscopic phenotypes.

Traditionally, statistical methods used to investigate biological basis of syndrome or biomedical conditions often focus on univariate methods such as Student's *t*-test and linear multivariate methods such as linear regression. These methods pay little attention to the complex associations between biological parameters in microcosmic level and syndrome. Advanced statistical methods, or called data mining methods, provide a better solution to establish the nonlinear association between syndrome and biological parameters in microcosmic levels.

**Figure 1:**
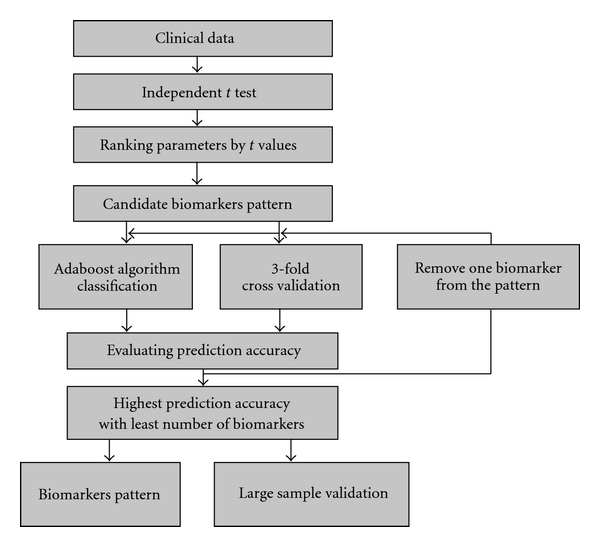
The flowchart of data mining scheme.

In this research, we presented a data mining-based strategy to establish diagnosis pattern of syndrome in TCM (Figure 1). We carried out clinical epidemiology surveys to collect macroscopic information and related microcosmic information. Based on the data and characteristic of it, we presented a novel data mining methods that combined the *t*-test with the Adaboost algorithm not only to choose as few as microcosmic biological parameters associated with QDS but also to establish a complex mathematic model for diagnosing QDS by using the selected parameters. The model had the ability to predict whether a UA patient is QDS. Moreover, we carried out a similar epidemiology survey with relatively large samples to test the accuracy and specificity of the established model. We also investigated and discussed the roles of selected parameters in UA and QDS. 

## 2. Methods

### 2.1. Materials

Fifty-two UA in-patients aged between 55 and 75 years came from Dongzhimen Hospital affiliated to Beijing University of Chinese Medicine and Hospital affiliated to Inner Mongolia Medical College (from 1 November 2006 to 31 March 2008). Thirty-nine UA patients with QDS and thirteen UA patients without QDS were included. The syndromes of a patient were judged by three TCM senior doctors with consistent diagnostic opinions according to diagnosis criteria established in 1990s in China (the appearance of all the following symptoms: lassitude and short breath, pale enlarged and tender-soft tongue possibly with teeth mark, sunken and fine pulse).

Eligible patients in this paper were defined as with UA based on diagnosis criteria of UA established in 2002 by ACC and AHA, i.e. chest pain at rest and transient S-T segment changes, without significant increases in creatine kinase and creatine kinase MB fraction [4]. The criterion for enrollment was admission within 48 h after the onset of chest pain. Moreover, the exclusion criteria were composed of four conditions: (i) besides UA, a patient also suffers from other cordis diseases such as acute myocardial infarction, myocarditis and cardiac nerve functional disease; (ii) a patient with angina caused by other diseases, for example rheumatic fever, syphilis, congenital coronary anomalies, hypertrophic cardiomyopathy, cardiac mitral stenosis; (iii) besides UA, a patient also suffers from stroke, diabetes, nephritis, renal failure, pulmonary infection, urinary tract infection, rheumatism, osteoarthritis, serious diseases caused by liver, renal, hematogenous system, incretion system; (iv) a woman patient in gestation or lactation.

All subjects gave informed consent and were approved by the Medical Ethics and Human Clinical Trial Committee at Dongzhimen Hospital or Hospital affiliated to Inner Mongolia Medical College.

It is noted that the health control group is not included in this study since we only investigate syndrome in the context of a disease. The control of UA with QDS is UA without QDS.

### 2.2. Biological Parameter Detection

Thirty-four biological parameters were detected on the 52 patients. The parameters included blood urea nitrogen (Bun), creatinine (Cr), uric acid (URA), total CO_2_, alanine aminotransferase (ALT), aspartate aminotransferase (AST), glutamyltransferase (GGT), total bilirubin (TBIL), direct bilirubin (DBIL), indirect bilirubin (IBIL), high density lipoprotein (HDL), low density lipoprotein (LDL), cholesterol (CHO), triglyceride (TG), white blood count (WBC), red blood count (RBC), hemoglobin (HGB), hematocrit (HCT), mean cell volume (MCV), mean corpuscular hemoglobin (MCH), mean corpuscular hemoglobin concentration (MCHC), platelet count (PLT), mean platelet volume (MPV), platelet distribution width (PDW), neutrophilic granulocyte absolute value (NE), leukomonocyte absolute value (LY), mononuclear cell absolute value (MO), acidophilic cell absolute value (EO) and basophilic cell absolute value (BA).

These parameters were divided into four parts: electrolyte, kidney and liver functions; blood routine and blood lipid. They were included as a full set based on the following two reasons. The first one is that they are very commonly used in clinical practice, which provides convenience for further validation and popularization. The second is that they are usually omitted in diagnosing syndrome in clinics nowadays. Less attention is paid on associations between them and syndromes, although they have some connections with the disease.

It is also noted that the association between biological parameters chosen here and the syndrome do not affect association between another parameters (such as inflammatory systems) and the syndrome. Since we only established a diagnosis avenue by several biological parameters for QDS, these several parameters are part, not all, of biological basis of the syndrome.

### 2.3. Data Mining Method: *T*-Test-Based Adaboost Algorithm

We used the Student's *t*-test to choose several specifications with possible significant difference between two subtypes and ranked the included specification by *t*-value calculated for them. The *t*-value is calculated as follows: $t=({\overset{̅}{X}}_{1}-{\overset{̅}{X}}_{2})/S_{{\overset{̅}{X}}_{1}-{\overset{̅}{X}}_{2}}$, *ν* = *n*_1_ − 1 + *n*_2_ − 1 = *n*_1_ + *n*_2_ − 2, where $S_{{\overset{̅}{X}}_{1}-{\overset{̅}{X}}_{2}}=\sqrt{S_{c}^{2}\left(1/n_{1}+1/n_{2}\right)},$ in which

(1) $$S_{c}^{2}=\frac{\left(n_{1}-1\right)S_{1}^{2}+\left(n_{2}-1\right)S_{2}^{2}}{n_{1}+n_{2}-2}=\frac{\sum {\left(X_{1}-{\overset{¯}{X}}_{1}\right)}^{2}+\sum {\left(X_{2}-{\overset{¯}{X}}_{2}\right)}^{2}}{n_{1}+n_{2}-2}.$$

Where ${\overset{̅}{X}}_{i}$, *i* = 1,2 denotes the mean value of a biological parameter *X*_*i*_  with QDS and non-QDS respectively. *ν* represents freedom of the distribution. Here, *n*_1_ = 39, *n*_2_ = 13. *S*_*i*_, *i* = 1,2 is responsible for standard derivation of the parameter *X*_*i*_  respectively.

The *t*-value of a biological parameter reflects the degree of difference between the QDS and the non-QDS group. The higher the value, the more significant the difference is between the two groups.

We used the *t*-test here to rank the biological parameters and formed a candidate pattern. The association between several parameters and QDS is established by classification models. Decision tree (DT) is a kind of classifications models and has been successfully applied in medical field [5]. Several research outcomes have demonstrated that DT combined with the boosting technique may improve classification accuracy and steadiness of the model [6]. Therefore, we used DT combined with the boosting technique as a classification and prediction model to build association between biological parameters and QDS here. AdaBoost, short for Adaptive Boosting, is a machine learning algorithm, formulated by Yoav Freund and Robert Schapire [7]. It is a kind of so-called meta-algorithms and it is embedded into classification methods to improve their predictive performance. AdaBoost is adaptive in the sense that subsequent classifiers built are tweaked in favor of those cases misclassified by previous classifiers.

AdaBoost is a classifier (Decision tree C4.5) [8] that is used to establish association between biological parameters and QDS repeatedly in a series of times, denoted as *t* = 1, 2,…, *N*. For each time a distribution of weights *W*_*t*_ is updated that indicates the importance of examples in the clinical epidemiology data for the classification. At each time, the weights of each incorrectly classified example are increased, so that the new classifier focuses more on those examples. A brief introduction of the Adaboost technique is given as follows.

Given: (*x*_1_, *y*_1_),…, (*x*_*M*_, *y*_*M*_), where *x*_*i*_ ∈ *X*, *y*_*i*_ ∈ *Y* = {1, −1}, *X* denotes biological parameters and *Y* denotes QDS, *Y* = 1 and *Y* = −1 mean that the case *i* is with QDS and non-QDS respectively. *M* represents the number of cases; here *M* = 52.

Initialize *W*_1_(*i*) = 1/*M*, for *t* = 1, 2,…, *N*. Find the classifier *C*_*t*_ : *X* → {+1, −1} that maximize the classification accuracy with respect to the distribution *W*_*t*_: *C*_*t*_ = argmin *β*_*j*__*C*_*j*_∈*C*_, where *β*_*j*_ = ∑_*i*=1_^*M*^ *W*_*t*_(*i*)[*y*_*i*_ ≠ *C*_*j*_].

Usually, the prerequisite of *β*_*t*_ is set as *β*_*t*_ < 0.5 otherwise the algorithm stops.

Choose a coefficient *α*_*t*_ = (1/2)ln ((1 − *β*_*t*_)/*β*_*t*_), where *β*_*t*_ is the weighted error rate of classifier *W*_*t*_.

The distribution is updated as *W*_*t*+1_(*i*) = *W*_*t*_(*i*)e^−*α*_*t*_*y*_*i*_*C*_*t*_(*x*_*i*_)^/*Z*_*t*_, where *Z*_*t*_ is a normalization factor that makes the updated *W*_*t*+1_(*i*) be a probability distribution.

The final classifier is the output as *C*(*x*) = sign(∑_*t*=1_^*N*^ *α*_*t*_ *C*_*t*_(*x*)) . Therefore, after selecting an optimal classifier *C*_*t*_ of the distribution *W*_*t*_, the examples *x*_*i*_ that the classifier *C*_*t*_ identified correctly are weighted less and those that identified incorrectly are weighted more. As a result, when the Adaboost algorithm is testing the performance of the distribution *W*_*t*+1_, it will select a classifier with better classification accuracy than its counterpart in the previous classifier missed [9, 10].

### 2.4. K-Fold Cross-validation

We used three performance measures of all classification methods: accuracy, sensitivity and specificity to evaluate the association built. A distinguished confusion matrix was obtained to calculate the three measures. The confusion matrix is a matrix representation of the classification results. The upper left cell denotes the number of samples classified as true while they were true (i.e. true positives, TP), and lower right cell denotes the number of samples classified as false while they were actually false (i.e. true false, TF). The other two cells (lower left cell and upper right cell) denote the number of samples misclassified. Specifically, the lower left cell denotes the number of samples classified as false while they were actually true (i.e. false negatives, FN), and the upper right cell denotes the number of samples classified as true while they were actually false (i.e. false positives, FP). Once the confusion matrixes were constructed, the accuracy, sensitivity and specificity are easily calculated as sensitivity = TP/(TP + FN); specificity = TN/(TN + FP); accuracy = (TP + TN)/(TP + FP + TN + FN), where TP, TN, FP and FN denote true positives, true negatives, false positives and false negatives, respectively (Table 1) [5].

**Figure 2:**
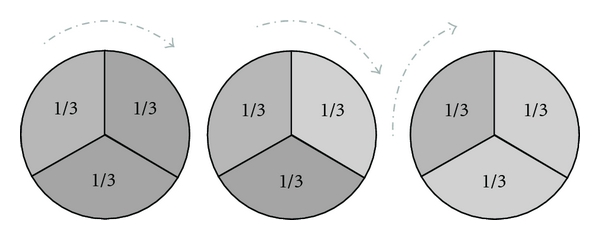
The illustration of a 3-fold cross validation technique.

A 3-fold cross validation was used here to minimize the bias produced by random sampling of the training and test data samples (Figure 2). 

The samples were equally divided into three parts. Each time, two parts were used to build the model and the other part was used to test the model. After three times, the confusion matrix was obtained and classification accuracy could be calculated.

## 3. Results

### 3.1. Data Mining

**Figure 3:**
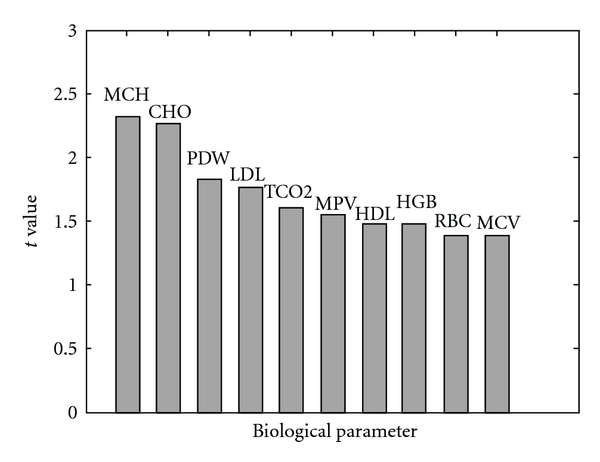
The absolute value of 10 biological parameters is given in a descending way.

**Figure 4:**
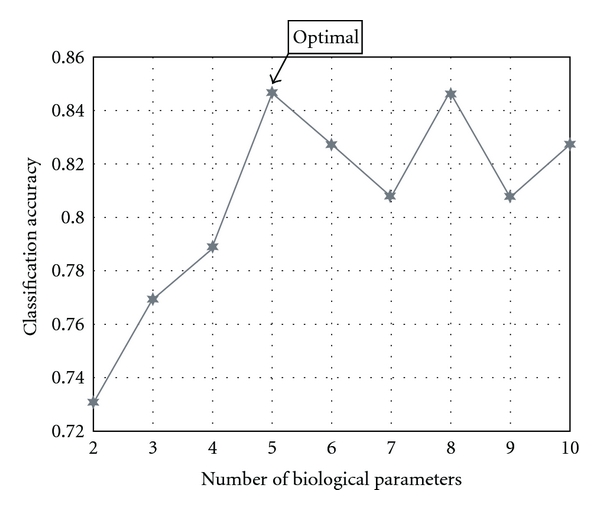
The *t*-test-based algorithm calculated different biological parameters combinations by nine times. The optimal number of biological parameters is five.

Significant difference was found in only two biological parameters (MCH and CHO) (*P* < 0.05) between unstable angina with QDS patients and the non-QDS patients. But the two biological parameters were not enough to distinguish the unstable angina with QDS patients from the non-QDS patients (Figure 4). So we chose 10 parameters to form a candidate subset from where the biological parameters pattern would be discovered by data mining methods (Figure 3). 

After the *t*-test based Adaboost algorithm was used, we got the three best patterns whose diagnosis accuracy of unstable angina with QDS could reach 84.5% (Figure 4). Considering the value of clinical application, the fewer indexes, the better; therefore, we prefer the pattern made by the five following parameters (Tables 2 and 3).

### 3.2. Clinical Validation

In order to test the accuracy of association established for QDS by five parameters. We re-carried out a clinical epidemiology survey to collect full information of both syndromes and five biological parameters. The inclusion and exclusion criteria were consistent with the former survey. Forty-two UA patients from hospital affiliated to Inner Mongolia Medical College in northern part of China and forty-three UA patients from hospital affiliated to Hubei college of Chinese medicine from in southern part of China were collected between June 2008 and March 2009 and were included to validate the model built by the *t*-test-based Adaboost algorithm. We input the five biological parameters into the prediction model. It would predict that if the case was QDS, then the prediction results were compared with diagnosis results by TCM experts, and the sensitivity, specificity and accuracy were calculated. As depicted in Table 4, it is found that the validation reached the accuracy of *∼*83%, which was in accordance with results obtained by a 3-fold cross-validation. These evidences showed that the five biological parameters did significantly associate with QDS and the prediction model had its conservation in different population and demographic area.

### 3.3. Association between Symptom That Related to QDS and Five Biological Parameters

Since QDS is diagnosed by the combination of symptoms as well as related tongue and pulse information, the association between QDS related symptoms and the five parameters needs to be investigated. We used full samples here with 137 UA patients to establish the association. According to the basic theories of TCM, hypodynamia is significantly associated with QDS. Using the same data mining method and a 3-fold cross-validation, it was found that the classification accuracy was *∼*80% (Table 5). 

## 4. Discussion

Nowadays, the diagnosis of syndromes in TCM mainly depends on four examinations (inspection, listening and smelling examination, inquiry and palpation). Thus, the accuracy is relatively low. If objective indexes can be used in the diagnosis of syndrome in TCM, the accuracy could be significantly improved.

During the past decades, the researches on the biological basis of diseases and TCM syndromes have encountered a bottleneck due to the direction of finding the “golden indicator” for the diagnosis of disease or syndrome. However, the syndrome is a holistic concept and its scientific content may need an overall review on organisms. Maybe there is no “golden indicator” for the diagnosis of a syndrome at all. The researches on syndromes should not be restricted in finding the “golden indicator.”

With the development of system biology, the data mining technique becomes more and more mature. Its advantages are very applicable to the complex correlativity study of macroscopic TCM symptom and microcosmic parameters. We had used decision tree as the data mining method to the study of the relation between symptom in TCM and biological parameter [11] and made a lot of progress.

In this paper, we took the *t*-test based Adaboost algorithm as a feature selection method to establish the diagnosis pattern of QDS in the context of UA for three reasons. The first one is that the algorithm is very simple to implement. The second one is that it performs feature selection on large sets of features. The final reason is that it is with fairly good generalization.

Among the five parameters, CHO and LDL have been proposed to be independent predictors of atherosclerotic vascular disease [12, 13]. CHO is an essential component of cell membranes where it is required to establish the proper permeability and fluidity of membrane. In addition, CHO is an important precursor molecule for the biosynthesis of bile acids, steroid hormones and several fat soluble vitamins [14]. But high CHO is one of the major risk factors for developing heart disease. LDL molecules are the major carriers of CHO in the blood. It is a type of lipoprotein that transports CHO and triglycerides from the liver to peripheral tissues. It has been accepted as an emerging cardiovascular risk factor by the National Cholesterol Education Program Adult Treatment Panel III [15]. LDL concentration is an important predictor of cardiovascular events and progression of coronary artery disease [16].

PDW may relate to QDS. PDW expresses the distribution of the size of platelets produced by the megakaryocyte. Although PDW have been available now for quite a long time, their clinical usefulness hitherto is not obvious. PDW has been found highly useful in the differential diagnosis of thrombocytosis [17]. Thrombocytosis relates with the blood stasis syndrome which always leads to QDS because, according to TCM, blood is the vehicle of Qi.

MCH and TCO_2_ may both associate with cardiovascular disease and QDS. MCH is the average mass of hemoglobin per red blood cell in a sample of blood. Hemoglobin is the iron-containing oxygen-transport metalloprotein in the red cells of the blood in human. Hemoglobin transports oxygen from the lungs to the rest of the body where it releases the oxygen. We need more oxygen and usually feel tired in sports. Insufficient oxygen transportation usually leads to short breath and lassitude, which are the typical symptoms of QDS. MCH might be a reflection of QDS. Hemoglobin also has a variety of other gas-transport and effect-modulation function. It has been reported to have association with heart failure [18]. TCO_2_ is a reflection of acid-base balance. Measurement of the total CO_2_ content can help us explain acid-base balance disorders. It is relative with the breath of lungs. Sports can increase respiratory frequency and cause tiredness, lassitude and short breath which are important characteristics of QDS. Thus, TCO_2_ appeared in our diagnosis pattern of QDS is reasonable. It has been reported that there is a relationship between breath obstacle and cardiovascular disease [19].

To sum up, the associated biomedical literatures support the finding. The five parameters are certainly ignored in clinics. However, we found that they are useful in the diagnosis pattern for UA with QDS.

In the pattern made by five biological parameters, *P* values of only two indexes (MCH and CHO) were <0.05. *P* values of PDW, LDL and TCO_2_ exceeded 0.05, which suggests that the three parameters has no significant difference between QDS and non-QDS respectively. However, combined and associated with the other two biological parameters, the classification model reaches a highest accuracy, which suggests that the five parameters have significant difference between the two groups.

So many documents have shown that inflammation system has significantly associated with UA and the syndromes in the context of it [11]. A syndrome called blood stasis syndrome was found to be diagnosed by several inflammation parameters. However, the association between QDS and inflammation system is not studied in the paper; the diagnosis accuracy of QDS by using biological parameters from inflammation system is need to be further investigated in our continuous research efforts and be compared with the results obtained in the paper.

The further research directions would focus on three parts. The first is to enlarge the amount of clinical epidemiology samples and use the data mining methods to establish diagnosis methods for all syndromes in the context of UA, i.e. differentiate diagnosis. Alternatively, the association of five biological parameters and QDS needs to be investigated by using animal models to study the inner mechanism of it. Finally, the diagnosis pattern should be studied in the context of other diseases than UA, which provides a basis for uncovering the biological basis of syndrome in TCM.

## 5. Conclusion

This study was devoted to establish the diagnosis method of QDS by using microcosmic biological parameters in the context of UA. We presented a novel data mining method to select five parameters: MCH, CHO, PDW, LDL and TCO_2_ to form a diagnosis pattern that distinguishes QDS from UA patients. The highest diagnosis accuracy of the patterns could reach 84.5%. The further clinical validation showed that the five parameters have significant association with QDS in the context of UA. The association between symptoms that are significantly associated with QDS also confirms the finding. We conclude that the syndrome in TCM can be diagnosed in the microcosmic level and the *t*-test-based Adaboost algorithm may have high value in diagnosis in the clinics.

## Funding

Grants from The National Basic Research Program of China (973 Program) (no. 2003CB517105); the National Department Public Benefit Research Foundation of China (no. 200807007); the Creation for Significant New Drugs Project of China (no. 2009ZX09502-018); National Natural Science Foundation of China (no. 30902020).

## Figures and Tables

**Table 1:** TP, TN, FP and FN of the pattern.

Feature selection method	TP	FN	FP	TN	Sensitivity (%)	Specificity (%)	Accuracy (%)
*T*-test based on Adaboost algorithm	35	4	5	8	89.7	61.5	84.5

**Table 2:** Using 52 samples, classification models reach an accuracy of 100%.

Samples	TP	FN	FP	TN	Sensitivity (%)	Specificity (%)	Accuracy (%)
Full samples	39	0	0	13	100	100	100

**Table 3:** The five indexes and their *P* value in the pattern included.

Biological parameter	*P*-value
MCH	0.023899
CHO	0.027355
PDW	0.072665
LDL	0.083111
TCO_2_	0.11374

**Table 4:** The clinically further validation of the association established: it is found that the results are in accordance with the cross-validation counterpart.

Region	Total	QDS	Non-QDS	TP	FN	FP	TN	Sensitivity (%)	Specificity (%)	Accuracy (%)
NeiMeng region	42	29	13	25	4	3	10	86.2	76.9	83.3
Hubei region	43	31	12	26	5	2	10	83.9	83.3	83.7
Total	85	60	25	51	9	5	20	85	80	83.5

**Table 5:** The association between symptom and the five biological parameters was established by the algorithm.

Samples	TP	FN	FP	TN	Sensitivity (%)	Specificity (%)	Accuracy (%)
Full samples	79	11	17	30	87.7	63.8	79.6

The classification accuracy is *∼*80%.
